# Beyond cDC1: Emerging Roles of DC Crosstalk in Cancer Immunity

**DOI:** 10.3389/fimmu.2019.01014

**Published:** 2019-05-09

**Authors:** Rajkumar Noubade, Sonia Majri-Morrison, Kristin V. Tarbell

**Affiliations:** Department of Inflammation and Oncology, Amgen Research, Amgen Inc., South San Francisco, CA, United States

**Keywords:** dendritic cells, cDC1, cDC2, crosstalk, cancer immunity

## Abstract

Dendritic cells (DCs) efficiently process and present antigens to T cells, and by integrating environmental signals, link innate and adaptive immunity. DCs also control the balance between tolerance and immunity, and are required for T-cell mediated anti-tumor immunity. One subset of classical DCs, cDC1, are particularly important for eliciting CD8 T cells that can kill tumor cells. cDC1s are superior in antigen cross-presentation, a process of presenting exogenous antigens on MHC class I to activate CD8^+^ T cells. Tumor-associated cDC1s can transport tumor antigen to the draining lymph node and cross-present tumor antigens, resulting in priming and activation of cytotoxic T cells. Although cross-presenting cDC1s are critical for eliciting anti-tumor T cell responses, the role and importance of other DC subsets in anti-tumor immunity is not as well-characterized. Recent literature in other contexts suggests that critical crosstalk between DC subsets can significantly alter biological outcomes, and these DC interactions likely also contribute significantly to tumor-specific immune responses. Therefore, antigen presentation by cDC1s may be necessary but not sufficient for maximal immune responses against cancer. Here, we discuss recent advances in the understanding of DC subset interactions to maximize anti-tumor immunity, and propose that such interactions should be considered for the development of better DC-targeted immunotherapies.

## Introduction

The interaction between various myeloid and lymphoid cell populations is crucial to initiate and orchestrate a robust anti-tumor response. By processing tumor associated antigens (TAAs) and migrating to draining lymph nodes (dLN), where T cell priming occurs, dendritic cells (DCs) are considered the most potent professional antigen presenting cells (APCs) to elicit adaptive anti-tumor immunity (1). In addition to presenting antigens, DCs use soluble molecules such as cytokines and chemokines as well as direct cell-cell contacts to prime and activate TAA-specific T cells. DCs were discovered by Ralph Steinman and Zanvil Cohn in 1973 as an APC population, distinct from macrophages, that initiate adaptive immune responses (2). As a result of more recent deep-phenotyping, DCs are now recognized to be a heterogenous population comprising several subsets distinguished by their development, phenotypic differences, localization, and functional specialization (2–6). This functional specialization of each subset allows DCs to initiate distinct immune responses in different immunological contexts (7). Here, we review literature supporting the hypothesis that, although one DC subset, conventional DC1(cDC1), has been shown to be crucial for anti-tumor immunity, multiple DC subsets, and interactions with other cells are needed for maximal responses.

### DC Subsets Are Functionally Specialized

DCs are broadly classified as classical (or conventional) DCs (cDCs) and plasmacytoid DCs (pDCs), each with specialized functions. cDCs, specialized in antigen presentation to naïve T cells can be further segregated into cDC1s and cDC2s, excelling in MHC class I- and class II-mediated antigen-presentation, respectively (3, 6, 8–10). cDCs are found both as lymphoid and non-lymphoid tissue cells, the latter of which can migrate via the lymph to dLN to present tissue-derived antigens (3, 11). cDC1s, present at lower frequency compared to cDC2s, are identified by the expression of XCR1 (12), and in humans, also by the expression of CD141 (BDCA3) (5, 13, 14). cDC1s possess specialized mechanisms to mediate efficient antigen recognition, antigen transport to appropriate endosomal compartments and subsequent processing for the presentation to CD8 T cells in a process known as cross-presentation (15–18). cDC1s can also activate CD4 T cells through MHC class II antigen presentation and can polarize activated CD4 T cells toward a Th1 phenotype through the secretion of IL-12 (19).

cDC2s are specialized in MHC class II-mediated antigen presentation and are the most efficient APCs for activation and expansion of CD4 T cells (5, 13, 20). They are the most frequent DC population present in blood, lymphoid organs and tissues and promote a wide range of immune responses including Th1, Th2, and Th17 in specific contexts (13, 19, 21–25). Human cDC2s can be identified by their preferential expression of CD1c (BDCA1) and CD172a (SIRPα) (26). cDC2s are more heterogenous than cDC1s, and express various receptors that enable them to respond to broad spectrum of microbial products (22, 26–28). A subset of Notch2-dependent cDC2s specializes in IL-23 production and contributes to innate defense and adaptive immune responses (27, 29).

pDCs, distinguished by their ability to produce large amounts of type I IFN upon viral infection (30–33) are identified, in humans, by the expression of surface markers CD303 (BDCA-2), CD304 (BDCA-4/Neuropilin) and CD123 (5, 13). They are present mainly in lymphoid organs and can migrate to the LN through blood circulation (5, 34). Mature pDCs can also act as APCs and have distinct regulation of MHC class II surface expression that results in sustained membrane peptide-MHC complex and antigen presentation (30). A heterogeneity of pDCs is also described in terms of their ability to produce type I IFN and/or antigen presentation (35, 36).

Another related but developmentally distinct population, derived from monocytes, termed monocyte DCs (moDC) upregulates certain functional properties of DCs in some contexts and express tumor necrosis factor (TNF)- α and intracellular nitric oxide synthase (iNOS) (37). More commonly, the term moDCs refers to monocyte isolated from human peripheral blood mononuclear cells (PBMC) that are *in vitro* differentiated in the presence of granulocyte-macrophage colony-stimulating factor (GM-CSF) and interleukin (IL)-4 into cells sharing several phenotypic and functional features of DCs (26, 38, 39). moDCs are the most common *in vitro* model of DCs, yet are quite heterogeneous in both mouse and human, with unclear relationship to *in vivo* cell populations (40–42).

All DC subsets, including cDCs and pDCs, are found in the tumor microenvironment (TME) (30, 43–47) and among the cDCs, the cDC2s outnumber cDC1s, with cDC1s being the rarest APCs within the TME (43, 48). The role of pDCs in tumor immunity remains elusive and contradictory. Similarly, the precise role of cDC2s in anti-tumor immunity has been difficult to delineate due to lack of proper genetic tools. On the contrary, mounting evidence suggests cDC1s to be the critical antigen presenting DC subset for the generation of anti-tumor immunity. Here we summarize data supporting the importance of cDC1s in anti-tumor immunity, and then review the recent literature that documents DC crosstalk being necessary for effective immune responses, in other contexts such as anti-viral immune responses, and apply these principles to tumor immunity.

### cDC1s Are Necessary for Anti-tumor Immunity

Since MHC class I molecules are expressed by every cell in the body (not just infected cells and cancer cells), to avoid bystander killing of healthy cells by CTLs, extracellular antigens do not enter the MHC class I-loading machinery (15, 18). Therefore, to generate an immune response, cancer cell antigens need special processing in APCs to be presented to naïve CD8 T cells. Moreover, naïve CD8 T cells primarily circulate through secondary lymphoid organs (15). Hence, cancer antigens must be brought to secondary lymphoid organs to be presented to naïve CD8 T cells. cDC1s fulfill both functions by patrolling tumor tissues, and by capturing, processing and presenting tumor-antigens on their surface through MHC class I molecules via antigen cross-presentation. cDC1s then migrate to dLN and deliver peptide/MHC class I signal to CD8 T cells which leads to their activation and the initiation of an immune response against tumor cells (15, 18).

Although other cell types have been reported to cross-present antigens (11, 49), this specialized function is mostly attributed to the cDC1 subset, owing to their unique adaptations of subcellular molecular machinery and vesicular trafficking (15, 18). Such adaptations include efficient antigen uptake of dying cells, delivery of cell-associated antigen to early endosomes, (15, 50–52), efficient phagosome-to-cytosol export of an ingested antigen possibly aided by ER-derived translocons and ER-associated degradation (ERAD) components such as Sec61, Derlin, p97 ATPase, Sec22 (15, 53–55), lower expression of lysosomal proteases (50) and antagonizing their degradative functions via NOX2-mediated ROS generation (56–60). The end result of such lower proteolysis, and therefore, increased antigen retention in cDC1s, is eventually an enhanced ability to carry the antigen all the way from peripheral tissues where the antigen is captured, to the dLN, where priming and activation of CD8 T cells occurs (56). The importance of cDC1s' ability to cross-present antigen in its immune functions is recently demonstrated using Wdfy4-deficient mice, which selectively lack cross-presentation (61).

Beyond their role in antigen cross-presentation, cDC1s are the major source of IL-12 production and thus influence anti-tumor immunity by activating NK cells and driving CD4 T cell responses toward Th1 responses (19, 62–64).

The critical role of cDC1s in anti-tumor immunity has been shown using mice deficient in basic leucine zipper transcription factor ATF-like 3 (Batf3), a transcription factor required for cDC1 differentiation (65). Batf3 knockout mice lack cDC1 cells but not other APCs and display impaired anti-tumor immunity in several models (43, 65–68). Expansion and activation of cDC1s using fms-related tyrosine kinase 3 ligand (Flt3L) and poly I:C leads to significant enhancement of antitumor responses (45). Immunotherapies such as PD1/PD-L1 blockade or CD137 agonists are ineffective in Batf3-deficient mice, highlighting the crucial role cDC1s in tumor immunity (68, 69). Furthermore, tumor-resident cDC1s are required for trafficking of adoptively transferred CD8 T cells into tumors through their ability to produce CXCL9 and CXCL10 (67, 70). DC-specific deletion of Sec22b leads not only to impaired cross-presentation of TAAs and reduced anti-tumor immune responses but also abolishes the efficacy of anti-PD1 therapy (53). In humans, the presence of cDC1s within the TME is associated with better prognosis and response to immunotherapy. Analysis of the cancer genome atlas (TCGA) dataset shows that a higher ratio of a cDC1 gene signature to a signature of all other myeloid cells (including monocyte/macrophage, and not just other DC subsets) is associated with better prognosis across human tumors (44, 71). Abundance of CD8 T cells positively correlates with cDC1 markers in pancreatic tumors (70). Taken together, these data show that cross-presenting cDC1s are crucial and necessary for the generation of an effective anti-tumor immunity.

### cDC1 Are Not Sufficient for Maximal Anti-tumor Immunity: Potential Roles of Other DC Subsets

**Figure 1:**
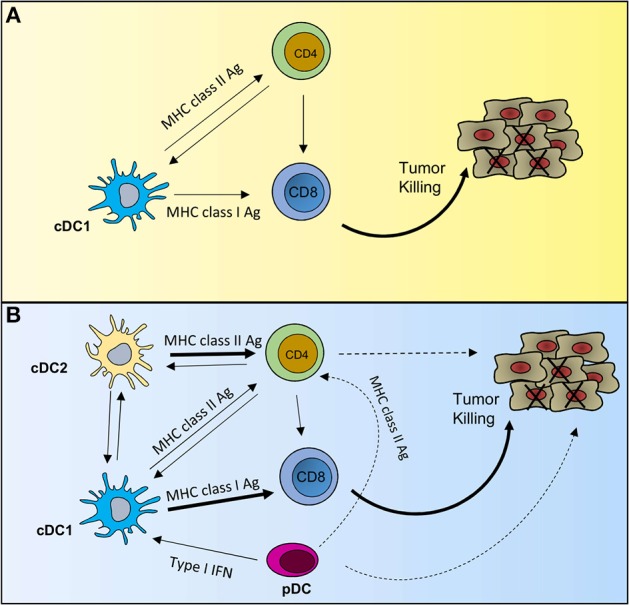
Potential scenarios of DC crosstalk in anti-tumor immunity. **(A)** Describes a scenario where an effective anti-tumor immune response would rely solely on cDC1 functions. cDC1s can activate both CD8 T cells and CD4 T cells through MHC class I- and MHC class II-mediated antigen presentation, respectively. Activated CD4 T cells provide licensing signal to cDC1s, which relay that help to CD8 T cells. Helped CD8 T cells have enhanced cytotoxic properties to efficiently kill tumor cells. **(B)** Describes multi-cellular interactions to achieve full-strength CTL responses against tumor. In this scenario, cDC1s predominantly activate CD8 T cells and cDC2s predominantly activate CD4 T cells. Activated CD4 T cells, in addition to providing help to maximize CTL responses can directly exhibit anti-tumor responses. Activated pDCs can modulate the TME mainly via type I IFN production, but can also activate CD4 T cells via MHC class II-mediated antigen presentation. Solid line indicates strong experimental evidence in tumor setting and dashed line indicates data in non-tumor setting. Thick line indicates predominant function.

Tumor immunology is built upon the tenet that cytotoxic CD8 T cells (CTLs) eliminate tumor cells (72) and the prevailing dogma is that cDC1s are the most potent APCs for the CTL response against tumor. Because of the strong evidence for the importance of cDC1 in tumor immunity, as presented in the previous section, in one scenario it is possible that cDC1s are the sole DC subset sufficient for optimal anti-tumor CTL generation through antigen presentation via MHC class I as well as MHC class II (Figure 1A). A major driver of the current dogma is the studies conducted in mice genetically manipulated to lack cDC1 such as Batf3-deficient or Zbtb46-Cre mice. However, these tools are imperfect. For example, Batf3 is expressed in cDC2 and effector CD4 T cells (65, 73) and Zbtb46 is also expressed in DC2s as well as in endothelial cells (74–76), raising the possibility of contributions from additional DC subsets and other cell types. Hence, it is not clear whether the cDC1 subset alone is sufficient to provide the maximal immunity against tumor. Recent evidence in non-tumor settings has demonstrated that cDC1s require significant contributions from other DC subsets and are not sufficient for an optimal CTL response (77–79), pointing toward a role for the other cells in shaping a robust and durable anti-tumor immunity.

Therefore, we describe a second scenario that includes possible roles of other DC subsets for a more robust anti-tumor immunity, directly and indirectly (Figure 1B). This scenario incorporates recent findings of spatiotemporal segregation of cDC1 and cDC2 activation within dLN to activate CD8 and CD4 T cells, respectively, during antiviral response. This robust CTL response requires interactions between multiple DC subsets, including cDCs and pDCs in a two-step priming process (77–79). Even though these responses are context dependent and are observed in anti-viral response, the general principals remain the same in anti-tumor immune response. Accordingly, in this scenario, the tumor-derived cDC1 primes CD8 T cells while tumor-derived cDC2 activates CD4 T cells in the first step of the CTL priming process and then in the second step, the activated CD4 T cells licenses a LN-resident cDC1 to relay the help for CTLs. Contributions of activated CD4 T cells to anti-tumor immunity can be more than just providing the help to CTLs, but also include activation of NK cells and macrophages through IFN-γ, modulation of tumor stroma and angiogenesis or direct cytolytic effects (80–83).

Additionally, during the two-step priming process, pDCs are recruited to cDC1-CD8 T cell priming sites, providing critical licensing signal to cDC1s through type 1 IFN. In this regard, lack of type 1 IFN receptor in cDC1s impairs their ability to reject tumors (84, 85). Furthermore, pDCs are usually weak APCs in the absence of activating signals but direct antigen presentation and T cell stimulation by pDCs has been described (30, 86). In fact, adoptive transfer of tumor-antigen-loaded pDCs induced potent anti-tumor T cell responses in melanoma patients (87), suggesting the possibility of anti-tumor immunity directly through APC functions by pDCs.

In the following sections, we mainly focus on this latter scenario of non-synchronous activation events by cDC1s and cDC2s and the reorganization of pDCs to the sites of CTL priming to describe the crosstalk between DC subsets and propose an integrated model of multi-DC subsets, multi-cell type interactions in achieving full-strength CTL responses in anti-tumor immunity.

### Crosstalk Between DC Subsets

One of the goals of cancer immunotherapy is to promote tumor-antigen specific T cell responses. The current data supports the notion that cDC1s are well-suited for this purpose and that they are usually necessary for the generation of an anti-tumor response. However, as discussed below, they may not be sufficient for full-strength anti-tumor cytotoxic T cell responses and interactions with other DC subsets contribute to this process. In the following sections, we will review the interactions between each DC subsets separately.

#### cDC-pDC Crosstalk

cDCs and pDCs are co-localized in many immune contexts, e.g., non-inflamed LNs, skin biopsies from lupus erythematosus patients, thyroid glands from autoimmune thyroiditis patients and spleens of cancer patients (88–90). Such close-proximity of pDCs and cDCs suggests possible functional coordination. Indeed, local production of type I IFN by pDCs induces stimulatory molecules on cDCs driving their maturation during an effective immune response (79). Intravital two-photon microscopic analysis of DC subsets within dLN during vaccinia virus infection showed active, CCR5-mediated recruitment of pDCs to the site of CD8 T cell priming by virus-infected cDC1. The activated CD8 T cells also orchestrate, via XCL1, recruitment of resident, non-infected XCR1^+^cDC1s. pDCs produce type I IFN to induce upregulation of costimulatory molecules including CD40, CD80, and CD86 on non-infected resident-cDC1s (79), driving their maturation and antigen-presentation functions leading to robust CTL response. pDC help for CTL response, either through type I IFN or other costimulatory molecules such as CD40L has been described in other viral infection models (91–93). Depletion of pDCs results in impaired CTL responses in many viral infections, e.g., VSV infection (94), LCMV infection (95), and cutaneous herpes simplex virus (HSV) (92). In the LCMV infection model, pDC-mediated CD4 T cell activation was essential in providing help and generation of anti-viral CTL response (95). These observations underscore the pivotal role of the crosstalk between DC subsets in maximizing immune response against cell-associated antigens.

Similarly, in the context of anti-tumor immune responses, cooperation between pDCs and cDC1s and the resulting synergistic effects dependent on soluble factors such as type I IFN and/or cell-cell contact between the two DC subsets are described (11, 47). The potent anti-tumor T cell responses induced in melanoma patients by adoptive transfer of tumor-antigen-loaded pDCs (87) could be either a result of direct priming by pDCs or via interactions with other cells, including cDCs. However, tumor infiltrating pDCs exhibit an abnormal or hypofunctional state, most likely due to immuno-suppressive effects of the TME such as TGFβ (96). The presence of pDCs in tumors is associated with poor prognosis in cancers such as breast and ovarian cancers (97, 98). pDCs are generally thought to contribute to tolerance induction and tumor promotion in this setting, most likely due to Treg induction and expression of immunosuppressive factors such as indoleamine 2,3-dioxygenase (IDO) (98, 99). Thus, the role of pDCs in shaping adaptive tumor immunity remains elusive. It likely depends on their activation status and involves cooperativity with other cells but how pDCs are activated needs further investigation.

#### cDC1-cDC2 Crosstalk

The two cDC subsets communicate not only through soluble mediators such as IL-12 but also through a third cell viz., activated CD4 T cell. Even though both cDC subsets are adept in priming naïve T cells, cDC2s are more proficient in activating CD4 T cells than CD8 T cells while cDC1s are potent activators of CD8 T cells but present antigen to CD4 T cells less efficiently, both *in vitro* and *in vivo* (8, 20, 43). However, recent literature demonstrates that robust and maximal induction of cytotoxic CD8 T cell responses against cell-associated antigens not only requires interactions with cDC1s, but also interactions involving cDC2s (77, 100). Intravital microscopy demonstrated that, in the dLN, the two cDC subsets exhibit differential localization wherein cDC1s are largely segregated to the T cell zone in deep paracortical regions and cDC2s are more peripherally distributed (78, 100–103) and that CD8 T cells cluster with cDC1s and CD4 T cells cluster with cDC2s during step one of two-step T cell priming event in anti-viral immunity (78, 100, 104), suggesting parallel activation of the two T cell subsets by two different cDCs in an asynchronous manner. Such differential localization of the cDC subsets into non-overlapping T cell regions is also reported in the spleen (105).

The peripheral DC subsets also exhibit different kinetics during their migration to dLN (106), with an implication that cDC2s might access CD4 T cells earlier. The CD4 T is cell activated in the first step of the priming process, then gets recruited to LN-resident, XCR1+ cDC1 during the second step of the priming process and delivers help signals to that cDC1. The receiver-cDC1 then transmits the help signal to CD8 T cell activated in the first step, resulting in a robust expansion of highly effective CTLs. In this regard, it is well-established that, in the absence of CD4 T cell help, CD8 T cell responses are weaker and insufficient to generate long-lasting memory (107–109). The CD4 T cell help includes molecules such as CD40L expressed on CD4 T cells, that induces expression of costimulatory molecules including CD70, CD80, CD86, and cytokines such as IL-12, IL-15 by cDC1 (66, 110–112). The molecular nature of CD4 T cell help in shaping the CTL response is recently reviewed (104) and will not be discussed here in detail. Signaling though type I IFN is critical for proper functioning of cDC1s (85) and cDC2s are one of the important sources of this cytokine, as shown by depletion of pDCs using anti-pDCA antibodies in Batf3-deficient mice (84).

cDC1s and cDC2s may also collaborate for optimal Th1 induction. In the context of leishmania infection, targeting antigen to either cDC1 or cDC2 can elicit IFNγ-producing T cells, but interestingly, the cDC2s require IL-12 produced by the cDC1s to induce Th1 responses, whereas the cDC1s induce Th1 responses via CD70, independent of IL-12 (19). Therefore, each DC subset provides different signals that can contribute to effector T cell responses. Among the activated CD4 T cells, Th1 cells excel in providing the help to cDC1s to prime and expand CTLs through of production large amounts of IFNγ (113), thus fostering an important crosstalk between the two cDCs.

The majority of the experimental data described above originates from studies in anti-viral immunity. However, where and how naïve cancer cell-specific CD4 T cells get activated in a tumor setting is less clear. Lessons learnt on the importance of MHC class II-restricted CD4 T cell responses in autoimmune pathogenesis may shed light on this question in anti-tumor responses as well, since the anti-tumor response is essentially a self-specific response (114). The highest genetic risk for autoimmunity is conferred by HLA class II genes, with odds ratios >6, suggesting that CD4 T cell responses are necessary for immunity against self. In the context of autoimmunity, although some priming in the target tissue may occur (115–117), most studies suggest that self-specific CD4 T cells are first primed in the dLN, suggesting that a similar phenomenon might be happening in the generation of an anti-tumor immune response.

### Evidence for the Importance of Tumor-Derived cDC2s and Activation of CD4 T Cells in the Draining Lymph Node

A large body of literature shows that naïve CD8 T cell activation for the generation of anti-tumor immunity occurs in dLN and is mediated by DCs (118–121). Interestingly, requirement of CD4 T cell help for optimal CD8 T cell effector functions in the context of tumor immunity is also well-documented, including the ability of CTLs to infiltrate the tumors (8, 119, 122–127). Non-helped CD8 T cells exhibit dysfunctional state with high expression of exhaustion markers in metastatic lung tumor model (127). In this regard, it is also well-established that the TME contains both cDC1 and cDC2 subsets (43–46). But importantly, both cDC1s and cDC2s scavenge tumor antigens (44) and migrate to dLN in a CCR7-dependent manner (46). Under right conditions, cDC2s can induce CD4 T cell activation in response to cell-associated antigen (51). Consistent with this, tumor-derived and dLN-derived cDC2s stimulate CD4 T cells more efficiently, *ex vivo*, in Lewis lung carcinoma model expressing ova as a model antigen (43). Furthermore, in this experimental setting, while cDC1 efficiently primed CD8 T cells, cDC2s are the most efficient activators of CD4 T cells. In addition, vaccination with the activated cDC2s reduced tumor growth, similar to that observed with cDC1s (43). Delivery of tumor antigen to cDC2 using dendritic cell immunoreceptor 2 (Dcir2) leads to significant anti-tumor effects in a mouse melanoma model (128). In a lung adenocarcinoma mouse model engineered to express MHC class II-restricted cytosolic antigen, activated cDC2 are observed both in the tumor and dLN and antigen-specific naïve CD4 T are activated in the dLN (129). In breast cancer patients gene signature of cDC2s positively correlates with better survival, similar to that observed with cDC1s, (130) and MHC class II expression predicts response to anti-PD1/PD-L1 therapy in melanoma patients (131). Collectively, tumor-derived cDC2s are likely to contribute to CD4 T cell activation in the dLN.

### Integrated Model of DC Crosstalk in Tumor Draining Lymph Node

**Figure 2:**
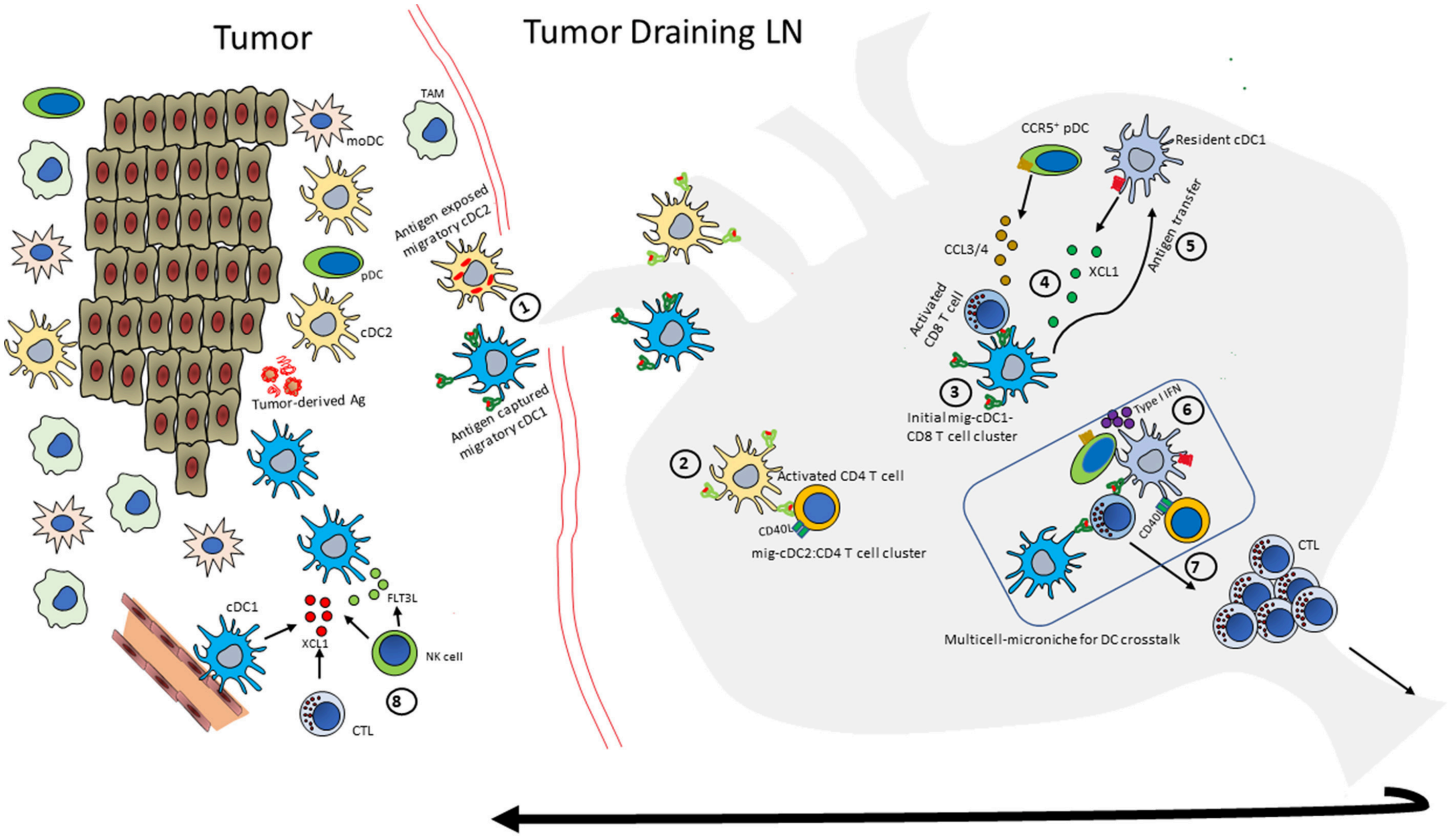
An integrated model of DC crosstalk for anti-tumor immunity. Outline of the multicellular orchestration of events that can contribute to a robust anti-tumor response. (Note: not all events happen in every context, and the order may also differ). (1) Intratumoral migratory-cDC1 and -cDC2s scavenge tumor-derived antigens and migrate to tumor dLN. (2) migratory-cDC2s (mig-cDC2) present MHC class II-restricted tumor antigen to CD4 T cells and induce expression of molecules such as CD40L (3) Migratory-cDC1s (mig-cDC1) prime and activate naïve CD8 T cells; (4) these activated CD8 T cells produce XCL1 and CCL3/4 to draw in XCR1+LN-resident-cDC1s and CCR5+ pDCs to the site of initial priming. (5) Mig-cDC1s can hand-off antigen to the newly recruited, LN-resident-cDC1. (6) pDCs produce type I IFN to mature cDCs. (7) The licensed CTL with enhanced effector functions undergoes clonal expansion and moves to the tumor to induce tumor cell killing. (8) The activated CD8 T cells and NK cells can mediate further increase in cDC1 numbers by producing XCL1 and FLT3L.

The spatiotemporal nature of DC crosstalk suggests two distinct DC-mediated events for maximal CD8 T cell responses: one after the initial antigen capture and another after the antigen is transferred to dLN-resident cDC1 cells (8). This sequential CTL activation is demonstrated by the exclusive clustering of migratory cDC1s with CD8 T cells early on during the initiation of an antiviral immune response. Subsequent clustering of activated CD8 T cells with the LN-resident cDC1s acts as a platform for signal relay from pDCs and activated CD4 T cells (79). According to this “consecutive interaction” model (79, 112), the generation of maximal CTL response and therapeutic anti-tumor immunity requires a multicellular orchestration of events in the tumor dLN (Figure 2) wherein migratory cDC1s capture the antigen in tumors, migrate to the dLN and form the initial priming site to activate CD8 T cells. The activated CD8 T cells produce CCL3/CCL4 and XCL1 to mediate recruitment of CCR5^+^pDCs and XCR1^+^LN-resident cDC1s, respectively. The migratory cDC1s handoff antigen to resident cDC1s in a yet-to-be-described mechanism (44, 106). In parallel, migratory cDC2s that have captured the antigen also move from the tumor to dLN and activate CD4 T cells. The pDCs induce the maturation of newly recruited, LN-resident cDC1s and the activated CD4 T cells licenses them for superior CTL responses. The overall effect of such orchestration and functional-cooperativity of pathways between different DC subsets is the amplification of CTL responses against a given antigen, without potentially missing out on the critical help necessary for CTLs to function at their peak. In fact, vaccine-mediated induction of such coordinated efforts of multiple DC subsets is known to trigger sustained and potent CTL responses while inhibiting immunosuppressive pathways in preclinical models (132). *Ex vivo* analyses of individual DC subsets might fail to identify such cellular orchestration to appreciate the relative contribution of each interaction between the different DC subsets in the generation of potent immune response.

### DC Crosstalk in Tumors *in situ*

Accumulating evidence suggest that cross-priming by tumor-resident cDC1 *in situ* is also an important phenomenon in the generation of an anti-tumor immune response. Local T cell priming and activation within tumors were observed in mice that lacked LN, or when T cell recirculation was blocked (133–135). Furthermore, intratumoral cDCs are required for the tumor regression achieved with adoptively transferred T cells in an experimental setting where migration of T cells to dLN was prevented (44). Moreover, tumor-resident cDC1s are the predominant sources of CXCL9 and CXCL10 and mediate recruitment effector T cells into the tumor (67). Similar to the events described for the dLN in the previous section, activated CD8 T cells could potentially orchestrate events *in situ* in the tumor where LN-like structures known as tertiary lymphoid structures (TLS) are present. A hallmark of TLS is the presence of high endothelial venules (HEVs) and expression of CCL19 and CCL21, the ligands for CCR7 (136, 137). DCs migrate in a CCR7-dependent manner (43, 45, 46, 138, 139). Moreover, well-organized TLSs contain B cell and T cells areas with mature DC subsets including cDCs and pDCs. Such organization makes TLS an ideal place to sustain proximity and the crosstalk between various subsets, and orchestrating local events required for maximal tumor immunity (135, 136). In fact, tumor-associated TLSs are functional structures capable of recruiting antigen-specific T cells and facilitating their activation through interactions with DCs (140). Interestingly, TLSs have been observed in several human tumors and their presence, particularly the ones containing high amounts of DCs and Th1 cells within the TLS, is associated with better prognosis (137, 141, 142) and increased TLS density is associated with strong infiltration of effector and memory CD8 T cells within the tumors (141), reflecting the importance of crosstalk between DC subsets and, CD4 help in increased CTL trafficking. Lung cancer patients with intratumoral CD8 T cells but no TLS had poor survival, indicating the necessity of their *in situ* education within the TLS for better effector functions (141, 143). In a metastatic lung tumor model, administration of TLR9 activator leads to CD8 T cell infiltration concurrent with TLS formation. The presence of TLS in this model was completely dependent on CD4 help (127). Taken together, these data suggest that TLSs promote DC crosstalk and anti-tumor immunity. Thus, induction of TLS provides another opportunity to promote communication between DC subsets to augment the magnitude of protective immunity, particularly against neoantigens that arise during the later phases of tumor progression (121). Moreover, induction of simultaneous trafficking and activation of cDCs and pDCs, using a vaccination strategy that combined DC subset-specific adjuvants (e.g., CpG-ODN and GM-CSF) leads to local accumulation of CD8 T cells and superior anti-tumor responses (132) suggesting that, even in the absence of TLS, evoking appropriate DC-crosstalk within the tumor tissue has the potential to boost superior CTL responses than targeting a single DC subset.

### Influence of DC Crosstalk With Other Cells in the TME on Anti-tumor Immunity

DCs can also engage with other immune cell types in the TME and lymphoid organs. Such interactions can enhance or dampen DC functions and anti-tumor immunity, depending on the cell types involved. For example, DCs interact with Treg cells, resulting in the suppression of CD8 T cell-mediated anti-tumor immunity (144). Two-photon laser-scanning microscopy analysis showed that Treg cells engage in prolonged physical interactions with DCs, six times longer than that of DC-CD8 T cell interaction in tumor. This extended physical contact between Treg cells and DCs results in upregulation of the immunosuppressive molecules such as IDO and lower maturation molecules on DC surface (144).

Interactions with other immune cell types such as natural killer (NK) cells with DCs can boost the immune response against tumors. It has long been established that, through the secretion of IL-12, cDC1s can license NK cells to kill tumor cells (145–147). However, recent studies have shown that NK cells can also influence DC functions in the context of tumors. In fact, NK cells produce XCL1 to recruit XCR^+^cDC1s to the TME (148). In addition, NK cells are one source of Flt3L within the tumor and dictate intratumoral accumulation of cDC1 cells by supporting DC survival, proliferation or development (71). Stimulation of NK cells with DC-derived factors such as IL-12, IL-15/IL-15Rα complex or contact–dependent interactions of OX40-OX40L augment NK cell functions to eliminate tumor cells (149–151). TCGA analysis suggests that NK cell/XCL1/cDC1 axis is associated with better survival in many cancer indications (148).

DCs also interact with NKT cells, the unconventional T lymphocytes expressing a semi-invariant T cell receptor (TCR) that recognize glycolipids presented by CD1d. (152). Although CD1d can be expressed by many hematopoietic cell types, DCs constitutively express CD1d and are the most potent APCs for exogenous glycolipids (153–155). The NKT cell ligand α-galactosylceramide (α-GalCer) acts as a potent *in vivo* adjuvant for DCs, resulting in increased expression of MHC class II and other costimulatory molecules (155). In addition, α-GalCer presented by DCs strongly activates NKT cells through CD40/CD40L interaction to induce IFN-γ production (156). Administration of α-GalCer was efficacious in preclinical tumor models (157) but not in patients (158), most likely due to soluble α-GalCer-induced anergy of NKT cell (159). Administration of α-GalCer, either soluble or loaded in DCs, is currently being explored to enhance anti-tumor immunity (160). Endogenous glycolipids are known to activate NKT cells (161) and CD1d expression is observed on tumor cells (162). In fact, the level of CD1d expression on tumor cells dictates NKT-mediated cytotoxicity (163).

Tumor-associated macrophages, in most carcinomas, are linked to poor prognosis primarily due to their immunosuppressive phenotype (164, 165). Macrophages produce IL-10 and in turn prevent IL-12 secretion of by DCs, resulting in dampened tumor-specific CD8 T cell activation (166). Among mononuclear phagocytes, monocyte-derived cells (including macrophages) are found at higher frequencies in tumors compared to DCs, and a higher monocyte-macrophage signature is associated with worse clinical prognosis (130, 167). These cells maintain a phenotype similar to *in vitro* M2 macrophages and contribute to the suppressive tumor microenvironment primarily via expression of anti-inflammatory mediators such as IL-10, TGF-β and IDO. Many of these signals dampen the ability of cDCs to present antigen in an immunogenic manner (164). However, in other contexts, macrophages can be inflammatory and effective APCs for eliciting T cell responses (168, 169). Thus, with the addition of the right signals, tumor macrophages have the potential to contribute to anti-tumor immunity.

Additionally, even though B cells have been described to play varied and often contrasting roles in the contexts of tumor immunity, emerging evidence suggests that B cells may also contribute to tumor immunity, both via antibody-mediated effects and by acting as APCs (170–172). Specifically, in terms of the crosstalk, DCs engage with B cells to promote their growth and differentiation, resulting in the production of antibodies. pDCs, through type I IFN production, can increase TLR7 expression and other activation markers on B cells (173). pDCs are specifically capable of inducing differentiation of activated B cells into Ig-secreting plasma cells through the secretion of type I IFN and IL-6 (174). Additionally, DCs dramatically enhance the secretion of IgG and IgA through the ligation of CD40 (175). B cells isolated from TLS-containing lung cancers showed significant antibody response against many TAAs (143, 176).

Finally, DC crosstalk with cancer cells has tremendous impact on the immune surveillance of the tumors. Cancer cells express several immunosuppressive factors such as PGE2, β-catenin and cytokines such as IL-10. PGE2 renders cDC1s unresponsive to XCL1 and CCL5 by downregulating *XCR1* and *CCR5* expression (148). β-catenin expression in cancer cells causes ATF3-mediated suppression of CCL4, the ligand for CCR5, leading to defective recruitment of cDC1 to the TME, and adversely affecting CD8 T cell priming against TAAs (177). Interestingly, PGE2 also induces the expression of β-catenin not only in tumor cells but also in stromal cells such as cancer associated fibroblasts (CAFs). CAFs respond to tumor-derived TNFα and IL-1β to secrete thymic stromal lymphopoietin (TSLP). TSLP is a strong driver of cDCs to activate Th2 CD4 T cells that are considered pro-tumorigenic (178). CAFs also produce stromal cell-derived factor 1 (SDF1) which drives cDCs toward tolerogenic DCs secreting IDO in a STAT3-dependent manner and promoting the recruitment and differentiation of Treg cells in tumors (179). However, co-targeting fibroblasts in combination with DC-based vaccine enhances the anti-tumor immune responses (180), suggesting that DC/stromal cell interactions can be manipulated to improve immunotherapies. Overall, with the property of bridging the innate and adaptive immune cells, DCs have a pivotal role in orchestrating an anti-tumor immune response by engaging interactions with many cell types within the TME.

### Potential Therapeutic Applications of Tumor DC-Crosstalk

The field of cancer immunotherapy, energized by the effect of T cell checkpoint inhibitors (CPI) in some patients, is beginning to focus on ways to treat “cold” tumors that lack T cells which can be activated with an anti-PD1 or other CPI. There is a large unmet medical need to increase the proportion of patients who respond to immunotherapy. Enhancing innate immunity, and DC function in particular, is one way to make tumors “warmer” that has tremendous potential. To date, most cell-based DC cancer therapies have utilized moDCs and have shown limited efficacy (121, 181, 182). With our current knowledge of both the importance of cross-presenting cDC1s for tumor immunity and the plasticity of monocyte-derived cells, moDCs are likely not the best cell type to use for inducing optimal clinical outcomes against cancer. Most studies show that moDCs have limited capacity for both cross-presentation and migration to draining LN compared to Batf3-dependent cells (43, 183). In addition, most monocyte-derived cells in the TME are immunosuppressive, and even if *ex vivo* moDCs can be activated to sustain cDC1-like properties, these are not likely maintained in the TME (121, 181). Therefore, moDC-based vaccines may not be the answer, and a new generation of DC-focused cancer immunotherapies are needed.

Increasing cDC1 function is one important goal, but as described here, some of this can occur indirectly via the cooperative interactions with other cells. In addition, both cDCs and pDCs have the potential to directly activate T cells that can kill cancer cells if exposed to the right activating signals (Figure 1B). Therefore, targeting maturation signals specifically to just cDC1s may not be the optimal therapy, and delivering signals that can enhance the function of all DC subsets may enhance efficacy and durability. For example, although tumor pDCs often correlate with poor prognosis, they are the most efficient producers of type 1 IFN and have the capacity for sustained MHC class II expression; these functions together may inflame the tumor and elicit strong T cell help that in turn could be sustained by newly matured cDC1s. Therefore, identifying signals that target and activate all DC subsets, and the cells that crosstalk with them will help provide novel insights into the cellular and molecular nature of tumor-specific CTL priming. The goal is to design therapies that build a site of sustained, immunostimulatory tumor-antigen presentation and increase the magnitude of anti-tumor immunity, so we can successfully treat a broader set of patients.

## Author Contributions

RN, SM-M, and KT designed and wrote the manuscript.

### Conflict of Interest Statement

RN, SM-M, and KT are full-time employees of Amgen Inc.
